# Preliminary Study on the Distribution, Source, and Ecological Risk of Typical Microplastics in Karst Groundwater in Guizhou Province, China

**DOI:** 10.3390/ijerph192214751

**Published:** 2022-11-10

**Authors:** Xianjin An, Wei Li, Jiacheng Lan, Muhammad Adnan

**Affiliations:** 1 School of Karst Science, Guizhou Normal University, Guiyang 550001, China; 2 State Engineering Technology Institute for Karst Desertification Control, Guiyang 550001, China; 3 College of Biology and Environmental Engineering, Guiyang University, Guiyang 550005, China; 4 State Key Laboratory of Environmental Geochemistry, Institute of Geochemistry, Chinese Academy of Sciences, Guiyang 550081, China; 5 University of Chinese Academy of Sciences, Beijing 100049, China

**Keywords:** karst, groundwater, microplastics, distributions, risk assessment

## Abstract

Karst groundwater is one of the important drinking water sources in karst areas, and it has an important role in maintaining the regional ecosystem and human health. The study of microplastics (MPs) in karst groundwater has rarely been reported, and the occurrence and migration behavior of MPs under the unique environmental conditions of karst is unclear. This study selected cave groundwater and common MPs in karst areas to explore the occurrence characteristics of MPs in groundwater to clarify the factors affecting the distribution and migration of MPs. The results showed that the abundance of MPs in karst groundwater was between 2.33 and 9.50 items·L^−1^, with an average abundance of 4.50 items·L^−1^. The microplastic size, type, color, and chemical composition were primarily 1~5 mm, film and fiber, color and transparent, and PS and PE, respectively. The risk characterization ratio (*RCR*) index results indicated that 80% of the samples were at a low ecological risk level, whereas 60% of the sampling points after concentrated rainfall in June were a medium ecological risk. The study showed that rainfall events significantly changed the abundance and migration of MPs in karst groundwater. The Pearson analysis showed a positive correlation between microplastic distribution and suspended particles (SP), total organic carbon (TOC), and water velocity (WV) in water. The study indicated that strong soil erosion in karst areas may also be one of the main sources of MPs in karst groundwater, and that karst groundwater microplastic pollution is an environmental problem that should not be ignored.

## 1. Introduction

Karst caves refer to natural underground spaces formed by the long-term dissolution, erosion, and gravity collapse of soluble rocks by groundwater [1]. It is an essential subsystem of the terrestrial natural environment, helping ensure the continued health of local ecosystems and the survival of the species that depend on it [2,3]. Due to a deficiency in surface water, karst groundwater is one of the region’s primary drinking water sources, and in fact, karst groundwater provides more than 70% of the drinking water in rural areas with dispersed water sources [4]. Karst cave water is special groundwater and an important part of regional water circulation; moreover, most karst caves in China lack effective protection [5], so the ability of karst groundwater to purify itself us relatively low and susceptible to outside pollutants. It is likely to become a natural enrichment place for all pollutants [6,7,8].

Microplastics (MPs) are plastic particles with a size of <5 mm, and a type of heterogeneous macromolecular polymer, which is a new concerning pollutant in the 21st century. In 2004, the concept of MPs was initially introduced to the scientific community [9]. MPs were smaller in size, similar to plankton, and easily swallowed by aquatic organisms. Its own bisphenol A, terephthalate, flame retardant, and coloring metals were toxic, which continuously accumulated within the organism, disturbed the stability of biological cells, and caused cytotoxicity [10,11], which would affect the normal physiological functioning of cells [12,13], destroy biological tissues and organs [14,15], and be transmitted step-by-step through the food chain [16]. China is a major producer and emitter of plastic waste, and the country’s soil, lakes, and groundwater are critically threatened by serious microplastic pollution [3,17,18]. MPs have a wide range of sources in natural water systems, including domestic sewage discharge [19], atmospheric settlement [20], agricultural irrigation [21], and sludge composting [22], etc. Karst area rural sewage discharge increased, and purification facilities were insufficient [23]. The discharge of sewage may have aggravated groundwater pollution in the karst environment. At the same time, soil agricultural film through surface runoff and karst cracks may have also become one of the main sources of regional water microplastic pollution. The agricultural mulching area reached 349,400 hectares in 2019 in Guizhou province, up 17.27% from 2018, and the usage of agricultural film reached 44,100 tons. Agricultural film recovery was more difficult in karst mountain soil. Due to the low recycling rate, plastic accumulation in soil and harm from microplastics in karst soil and water environments may be more significant.

MPs in soil [24,25], oceans [26], lakes [27], atmosphere [28], sludge [29] ecosystems and plants [30], animals [31], microorganisms [32], and humans [33] have been widely studied. MPs are considered to be widespread pollutants in the atmosphere, with a size of 15–250 μm, 1–2 orders of magnitude higher than other types of atmospheric aerosols that are typically less than 2.5 μm in diameter, and because of their small size, low density, easy entrainment, and long-distance transport, they present a threat to human health [34]. Microplastic pollution in bottled drinking water is <1 items·m^−3^ [35]. The detrimental effects of MPs on crop development and soil health have been reported. The crop-soil animal properties might decrease with increased time and microplastics over 10,000 mg·kg^−1^, the crop-soil properties might decrease with increased time and macroplastics over 240 kg·ha^−1^ [36]. MPs were discovered to have a clear enrichment impact on aromatic hydrocarbons, and compounds enriched in aromatic hydrocarbons were found to be human carcinogens [37]. However, the effect of MPs on some receptors was still controversial. For example, a study found that modest dosages of polyethylene MPs did not alter the physical and chemical properties of soil at a high-density [38]. In the short term, field testing of pure polyethylene microplastic exhibited no discernible effect on soil biomass and function [39]. At the same time, as a unique and important ecosystem, MPs have been studied in fragile karst environments, less frequently, with most studies conducted in relatively closed caves with environmental conditions. It has been established by several studies that MPs are dispersed in karst regions and that their distribution is connected to karst features. Plastic products in the high-altitude environment are becoming a new pollution problem; a study that examined the correlation between the physicochemical features of karst surface water and feed of livestock showed the presence of MPs in the stomachs of livestock [40]. It was determined that the greatest abundance of MPs in karst groundwater was 15.2 items·L^−1^; all MPs were fibers, by studying their structure; and the main sources of these contaminants were 14 karst springs and 3 wells in the karst region [41]. The study also showed that septic tank sewage may be the main source of microplastic pollution in the region. Characteristics of microplastic pollution in the sediment of an Italian tourist cave was studied for the first time [42], where it was found that there were 4390 items·kg^−1^ in the sediment of the cave’s underground river and only 1600 items·kg^−1^ in the surrounding non-tourist area. Therefore, the characteristics of MP occurrence in karst cave groundwater has not been fully studied, and a more thorough, comprehensive, and detailed investigation is necessary. It is especially imperative to understand the impacts of MPs underground and their risk to the water environment.

The factors influencing the environmental behavior of MPs are complex and diverse. The migration of MPs in soil, water, and other environmental media is closely related to the characteristics of the media, properties, and structure of the MPs, but also closely related to the surrounding environmental conditions. However, the negative effect of MPs on plant soil in silt loam may be obscured by the fact that global warming has a bigger negative influence on plant health than MPs [43]. The antagonistic effects between MPs and warming may enhance the tolerance of red tide algae species to microplastic stress, and warming reduces the detrimental effect of low MPs on algal cells and increases the inhibitory effect of high concentrations of MPs on algal cells [44]. Microplastic abundance in seawater was shown to be relatively unaffected by the frequency of tropical cyclones, as reported by [26]. The unique karst environment changes according to the different seasons, affecting the dissolved calcium in soil and water, water temperature, water flow rate, and flow density [45]. However, the impact of these factors on MP migration behavior in karst groundwater remains unclear.

We investigated MPs in karst cave groundwater in Guizhou province to study the spatial occurrence of MPs in the cave water of karst and the influence of karst environmental factors on the occurrence and migration of MPs, analyze the main sources of MPs in a karst cave, and study the ecological risk of MPs in cave groundwater. We clarified the karst cave groundwater system of microplastic pollution and migration and provided a scientific measure of microplastic occurrence, spatial distribution, support migration mechanism, and ecological environment impact. Our findings have great significance for regional ecological security, resident health, and the comprehensive prevention and control of groundwater pollution.

## 2. Materials and Methods

### 2.1. The Study Area

The underground river water-sediment of the Dragon Palace, a typical karst tourist cave in Xixiu District, Anshun, Guizhou Province, was selected as the study focus. The Dragon Palace is located in the southern suburbs of Anshun (North latitude 25°21′–26°38′, East longitude 105°13′–106°34′), located in the subtropical monsoon zone, with a humid climate and annual temperature of 14°. The annual rainfall of the Xixiu District was 1707.8 mm in 2020 (data from Anshun in 2020, Guoming Economic and Social Development Statistical Bulletin), and its elevation is about 1200~1300 m. The total area is about 60 km^2^ and the Dragon Palace river basin area is about 250 km^2^, located upstream of Wanger River, a tributary of the Pearl River system. Most of the groundwater system originates from the Anshun Lysogen watershed, from north to south, to the Dragon Palace water cave as it exits into the Wanger River of Beipan River. The Dragon Palace is a national 5A tourist attraction in Guizhou Province, with a high density of people during the peak season (March to November). The total length of the Dragon Palace underground river is about 15,000 m (with 5 sections in total, and the first 3 sections open to the public), and 1200 m sections (the first two sections) have been opened for tourism. The widest point of the underground river is 30 m, the narrowest is 2 m, with an average water depth of 26 m and a flow rate of 2~48 m³·s^−1^.

### 2.2. Groundwater Samples

**Figure 1:**
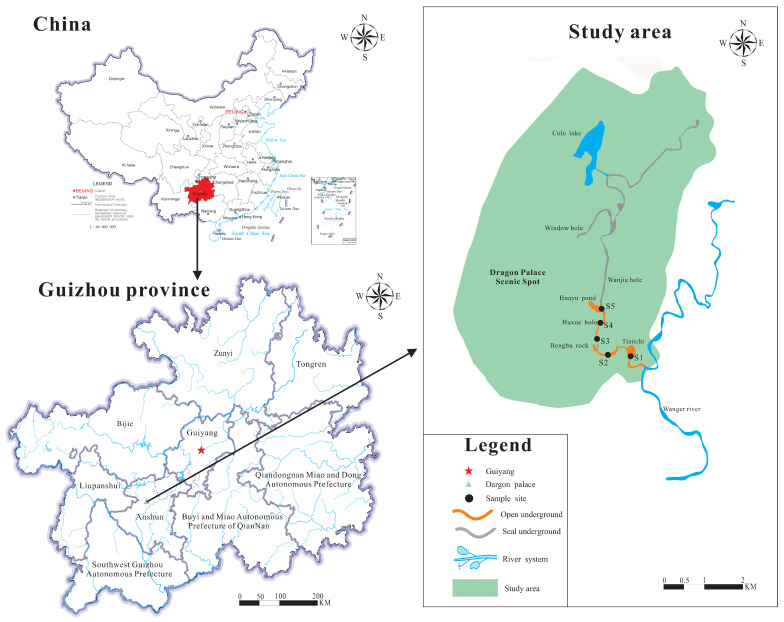
Distribution of groundwater sampling sites.

The volume of surface water collected corresponded to the monthly rainfall at the sampling site; the surface water was collected on 30 March 2021 (10.4 mm in March), 30 June 2021 (238.1 mm in June), and 31 October 2021 (68.0 mm in October). The surface water was collected from five sampling locations, as shown in Figure 1. Three parallel water samples were simultaneously collected from each sampling site. Our sampling method involved using an organic glass 2 L type water collector to collect the upper water samples at 10 cm from the water surface, placed them in a brown glass bottle and returned to the laboratory as soon as possible, and stored them at 4 °C for further analysis. A portable conventional five-parameter water quality detector JC-607 (Qingdao Jingcheng instrument co., LTD) was used to determine water pH, water temperature (WT), electrical conductivity (EC), and suspended particles (SP) with resolutions of 0.01, 0.01°, 0.01 μS·cm^−1^, and 0.1 mg·L^−1^, respectively. Total dissoluble solid (TDS) was measured using a CON200 tester (SENBE instruments co. LTD), with a resolution of 0.01 mg·L^−1^; total organic carbon (TOC) was measured using an elementary total organic carbon analyzer with a resolution of 0.01 mg·L^−1^; a portable flow meter XY-JCM2 (Qingdao lingheng environmental technology company) was used to detect water velocity (WV) with a resolution of 0.01 m·s^−1^, as shown in Table 1.

### 2.3. Quantitative and Qualitative Characterization of MPs

According to [16], the size of MPs was defined as between 1 μm and 5 mm. In the study, the sampled water was filtered by a 1 μm stainless steel filter, then the screen was washed with deionized water, and the trapped residue was washed in a glass container, sealed with ice back to the lab, and stored at 4 °C. Subsequently, the water samples in the glass container were transferred through a 1 μm filter film (Whatman), rinsed with deionized water to a 1 L beaker, and added with 200 mL of 30% H_2_O_2_ for digestion at 22 °C for 24 h. Then, 800 mL of saturated NaCl solution was added, fully stirred, and placed for 24 h. The supernatant was then collected through 1 μm filter film in the laboratory, and the trapped material was placed in a clean glass dish for testing.

**Figure 2:**
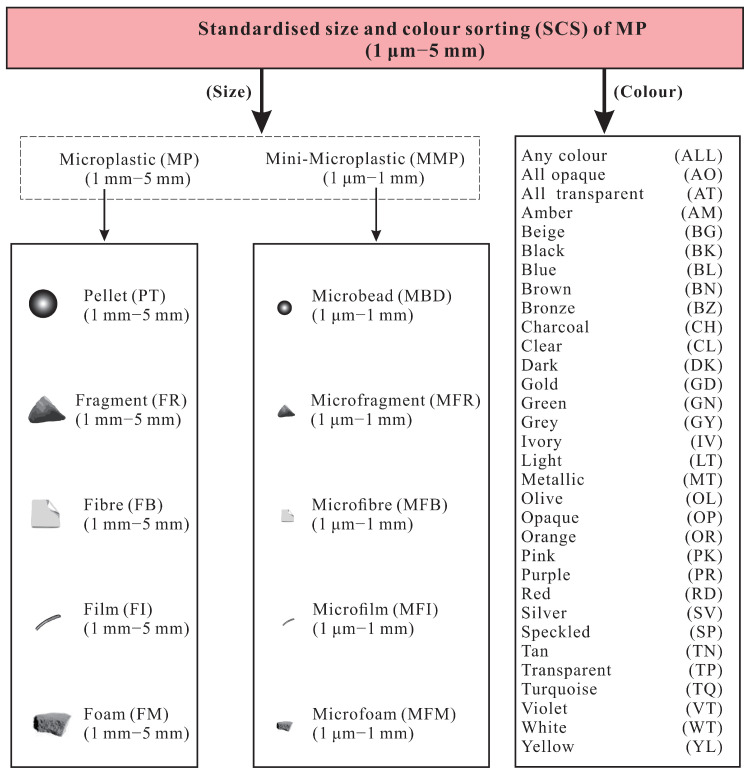
The standardized size and color sorting (SCS) system.

Water filtration flotation was measured with a handheld electron microscope (Dino-Lite AM3011T) with Dino Capture 2.0 software, and MPs were measured by Image J software (1.46r, National Institutes of Health, Bethesda, MA, USA) and classified according to the standard size and color classification system (SCS), as shown in Figure 2. We used a μ-Roman spectrometer for qualitative analysis of MPs and compared the standard spectra of six typical plastics (PA, PS, PP, PP, PE, PET, and PVC). When matching degree ≥70%, the material was identified as this type of plastic. Non-six typical plastics were classified as other component MPs.

### 2.4. Ecological Risk of MPs

The ecological risk of MPs in karst groundwater was the appearance of different polymer MPs. Therefore, this study used the polymer hazard index to calculate the polymer risk index *H* value of MPs and defined the *H* hazard level [46], as shown in Table 2. Meanwhile, the risk characterization ratio *RCR* was used to evaluate the ecological risk to calculate the degree of pollution caused by MPs in the ecological environment. Finally, four pollution levels were designated according to the *RCR*, as shown in Table 3. The *RCR* calculation formula is as follows:

(1) $$H=\sum P_{i}\cdot Z_{i}$$

(2) $$MEC_{z}=MEC\cdot H$$

(3) $$RCR=MEC_{z}∕PNEC$$

In Equation (1), *H* represents the polymer risk index caused by MPs, *Pi* represents the proportion of *i* plastic in the measured environmental concentration, and *Zi* represents the adjusted hazard index of *i* plastic. The *Zi* values of MPs polystyrene (PS), polypropylene (PP), polyethylene (PE), polyamide (PA), polyethylene terephthalate (PET), and polyvinyl chloride (PVC) were 2, 0.07, 0.73, 3.33, 0.27, and 666.73 [27]. In Equation (2), *MEC* represents the measured concentration of the MPs at each sampling site, and *MECz* represents the measured concentration of the adjusted MPs. In Equation (3), *RCR* represents the risk characterization ratio of MPs at each sampling site, and *PNEC* represents the predicted no-effect concentration by the species sensitivity distribution method. In this study, the underground water *PNEC* value of 4920 items·m^−3^ was used as the reference value, which was calculated by [47].

### 2.5. Quality Control and Data Analysis

In the sampling process of karst groundwater in Guizhou province, a wet 20 μm aperture filter film was placed in a clean culture dish near the sampling point as the environmental background value. Before laboratory analysis, the experimenter wore a cotton suit, mask, and nitrile gloves, wiped the experiment table with 70% alcohol, and filtered a glass fiber filter (Whatman, GF/F). Three indoor environmental pollution background controls were set in the experiment, and the container was covered with tin foil. All the final sample abundance values were corrected with the experimental background values. Microplastic abundance in water was represented using the unit items·L^−1^, and the results were plotted using OriginPro 2021b and analyzed for significance test using SPSS software, with *p* < 0.05 representing statistically significant differences.

## 3. Results and Analysis

### 3.1. Abundance of MPs in Karst Groundwater

The 405 MPs in 15 water samples were detected, as shown in Table 4, indicating that MPs are ubiquitous in the karst groundwater of Dragon Palace. The abundance of MPs was between 2.33 and 9.50 items·L^−1^, with an average abundance of 4.50 items·L^−1^. Analysis from the sampling time showed that the microplastic abundance of groundwater samples collected on June 30th was significantly higher than that on March 30th and October 31st. From the geographical location analysis of the sampling sites, the abundances of S1 (6.00 items·L^−1^) and S5 (5.33 items·L^−1^) were significantly greater than those of the other three sampling locations, with average values of 4.33 items·L^−1^, 3.72 items·L^−1^ and 3.11 items·L^−1^, respectively; S4 sampling sites had the least abundance.

### 3.2. Type, Size, and Colors of MPs

**Figure 3:**
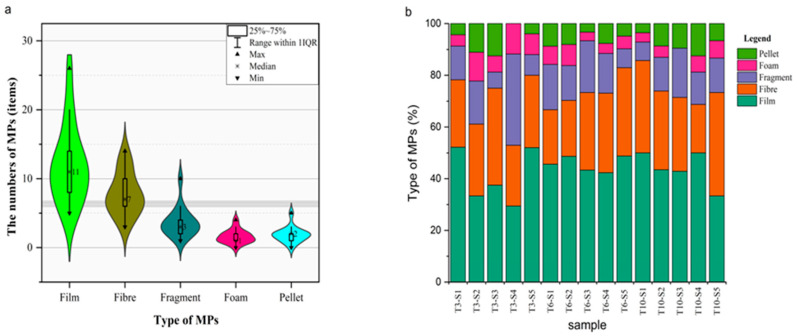
The type of MPs: (**a**) abundance of MPs in different shapes and (**b**) proportion of microplastic type.

The main types of MPs in the study area were film, fiber, foam, fragment, and pellet in the 15 water samples. The results showed that 181 film MPs in the 405 MPs accounted for the highest proportion of 44.69%, fiber was 28.89%, and fragment, pellet, and foam MPs accounted for 13.58, 6.91, and 5.93%, respectively, as shown in (Figure 3a,b).

**Figure 4:**
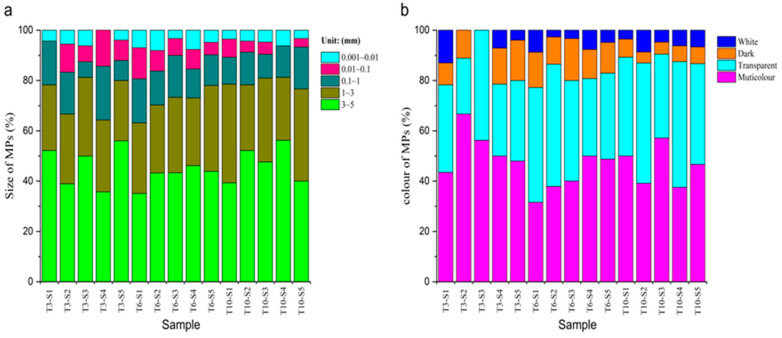
The proportion of size and color of MPs: (**a**) size and (**b**) color.

In the 15 water samples, the size of MPs was mainly 3~5 mm, accounting for 44.20%, and the number of MPs gradually decreased for sizes of 1~3, 0.1~1, 0.01~0.1, and 0.001~0.01 mm accounting for 29.88, 13.83, 6.91, and 5.19%, respectively, as shown in Figure 4a. This indicated the gradual decomposition of large-sized plastics to small-sized MPs.

The karst groundwater samples contained MPs in four color categories, namely, transparent, white, black, and colored (predominantly red, blue, brown, and yellow). Colored MPs accounted for the highest proportion, with 44.94%, followed by transparent MPs, accounting for 39.01%, and black and white MPs accounting for 10.37 and 5.68%, respectively, as shown in (Figure 4b).

### 3.3. Microplastic Components

**Figure 5:**
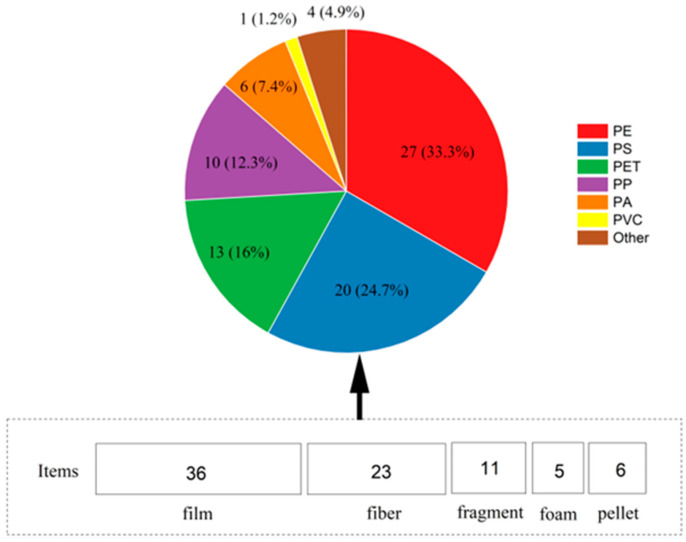
Microplastic polymers in 81 analysis samples by μ-Roman.

In the study, 81 samples of MPs of different types from 15 water samples (including 36 film, 23 fiber, 11 fragment, 5 foam, and 6 pellet samples) were randomly selected for μ-Roman determination. The results showed that the main components of microplastic films in karst groundwater were PE, followed by PS and PET; the main fiber polymers were PET, PA, and PP; fragments included PS and PA, foam was PS, and pellet was primarily PP and PE. The measurement results showed that out of the 81 microplastic samples, 4 other MPs were used, accounting for 4.94%; their main components were polyurethane and styrene butadiene rubber, as shown in (Figure 5).

### 3.4. Ecological Risk of MPs in Karst Underground Water

**Figure 6:**
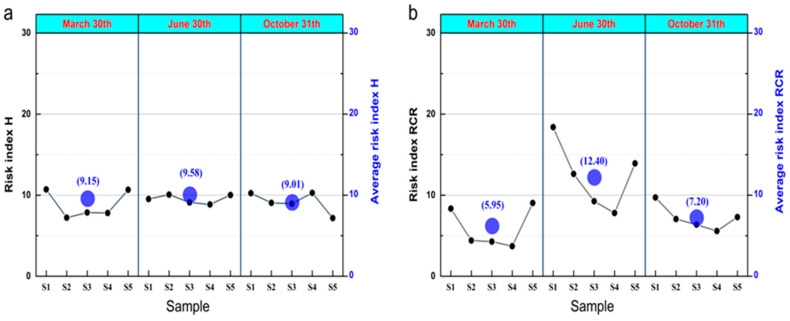
The risk value *H* and *RCR* of MPs in groundwater: (**a**) *H* risk index and (**b**) *RCR* risk index.

The ecological risk of MPs in underground karst water is crucial for living organisms and drinking water safety. As we only accurately determined 20% of the MPs, we could not accurately represent the degree of ecological risk in the study area; however, it had practical reference significance. The study ignored the risk of other polymers because microplastic polymers may form more consistent plastic types under the same environmental conditions; also, other polymers made up less than 5%. Therefore, the number of statistical inversion polymers for all the types of MPs at the three sampling time points was studied to calculate the ecological risk value of karst groundwater at the three-time points. The study results showed that 60% of MPs had an *H* risk grade of grade II in the 15 water samples, and the *H* value ranged between 7.23 and 10.72. From the average of the three sampling time sites, the highest *H* risk value was 9.58 on 30 June, with a rainwater concentration of 238.1 mm, which was slightly higher than the polymer risk level on 30 March (9.15) and 31 October (9.01), as shown in Figure 6a. The risk characterization ratio showed moderate ecological risk in the S1, S2, and S5 locations sampled in June. The *RCR* risk grade of the remaining water samples was a slight ecological risk, with *RCR* values between 3.70 and 18.39. From the three sampling time points, the *RCR* risk value of MPs in June of the concentrated rainwater was the highest, at 12.40, significantly higher than the *RCR* risk level in March (5.95) and October (7.20), as shown in Figure 6b. The risk index *H* of MPs in groundwater did not significantly change under different rainfall conditions. In contrast, the ecological risk level *RCR* significantly increased with an increase in rainfall.

## 4. Discussion

### 4.1. Microplastic Occurrence and Source Specificity in Karst Groundwater

**Figure 7:**
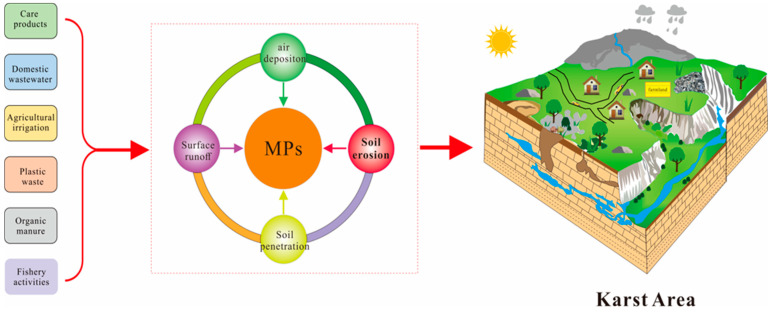
Schematic diagram of the main sources of MPs in karst groundwater.

Environmental MPs are emerging pollutants whose occurrence and migration behavior significantly impacts the ecosystem and animal and plant health. The research on MPs in freshwater in China has mainly concentrated on the Yangtze River basin, Pearl River basin, and some urban inland waters. With increasing human demand for freshwater, the pollution of freshwater by MPs has attracted attention worldwide. The pollution abundance of MPs in the freshwater environment greatly varies. The average abundance of floating MPs in the Great Lakes drainage basin was up to 43,000 items·km^−2^ [48]. The surface water of geographically remote and sparsely populated lakes in northern Mongolia also had microplastic pollution, with an average abundance of 20,264 items·km^−2^ [49]. The microplastic pollution of urban lakes and rivers in Wuhan was revealed by [50]. The highest pollution concentration in the northern lakes was 8925 ± 1591 items·m^−3^ and showed a significant positive correlation between pollution abundance and population density. Taihu Lake is an important freshwater lake in China, with a pollution abundance of 3.4~25.8 items·L^−1^, and floating MPs reaching up to 6.8 million items·km^−2^. According to [51], the pollution concentration of MPs in the freshwater environment considerably varies from one location to another, with an abundance of MPs as high as 34.1 × 10^5^~136 × 10^5^ items·km^−2^ in the main stream of the Yangtze River, near the Three Gorges Dam. This study showed that the karst groundwater microplastic pollution abundance was between 2.33 and 9.50 items·L^−1^, with an average abundance of 4.50 items·L^−1^, which is higher than the national average of 3.60 ± 5.92 items·L^−1^ [27]. The abundance of microplastic pollution in karst groundwater was similar to that of the 4.70 items·L^−1^ in the Three Gorges reservoir. It is evident that microplastic pollution in karst groundwater is an environmental problem that cannot be ignored in karst areas. Karst area underground water is the main source of surface lake water, mainly appearing as atmospheric transport, surface runoff, wastewater, agricultural irrigation, etc. In particular, a karst region that experienced heavy soil erosion was characterized by a high degree of spatial complexity, rapid increase in rock fissure height, and extensive utilization of the region’s agricultural potential. Figure 7 shows that the main source of microplastic contamination in karst underground rivers is the transmission of microplastic from farms through fissures.

This study has shown that the most important MP type of karst groundwater was film, which accounted for 44.69% of total MPs. Our results had some differences from [27], who showed that land surface water fiber was the main pollution type, accounting for 58%. This may be related to the use of massive agricultural film and packaging bags in karst areas [52]. The sampling site of this study was a grade 5A scenic area that may be associated with the use of a large number of artificial plastic packaging bags. The results showed that 28.89% of fiber MPs were also an important type of karst groundwater MPs. Previous studies have shown that fiber MPs mainly come from wash wastewater, suspended atmospheric particles, tire wear particles, and waste products generated from fishing activities [25]. The centralized treatment level of domestic wastewater in karst areas is relatively low. Some farmers still wash their clothes and perform other living habits in the river, which are a significant source of microplastic pollution in karst groundwater fiber. This has some differences from fibrous MPs in groundwater surface runoff, agriculture, and atmospheric subsidence in non-karst areas.

The common size of MPs in the surface water of China is <1 mm [27]. However, karst underground river MPs were mainly 1~5 mm in our study, accounting for 74.08% of total MPs. The size of the karst underground river MPs was obviously different from sizes distributed in other surface water bodies, which may be closely related to differences in environmental conditions. Despite the complex changes in natural conditions in karst areas, the long-term migration of MPs in the underground environment leads to less accepted environmental effects, which may be the main reason for the concentration of large-sized MPs in karst underground rivers. At the same time, there is a need to pay attention to small size microplastic, such as <1 mm microplastic, as one of its main sources are personal care products, especially the use of skin care products [53], and the economic level of the karst area is relatively low. There was likely less use of personal skin care products, which would also contribute to low quantities of small-sized MPs and beads pellet type microplastic in the karst groundwater.

The composition of MPs in karst underground rivers was mainly PE-type polymers, accounting for 33.33%, followed by PS and PET-type MPs, accounting for 24.69 and 16.05%, respectively. Most PE and PS are widely used in daily life [54], whereas the main source of PET is clothing products [55]. The most common microplastic polymers in the lower Yangtze River are PE, PS, and PP [56]. PE is the main component of agricultural plastic film [57], which is also consistent with the enrichment of groundwater with thin film microplastic pollution caused by soil erosion in karst areas.

### 4.2. Effects of Environmental Factors on Distribution and Transport of MPs

**Figure 8:**
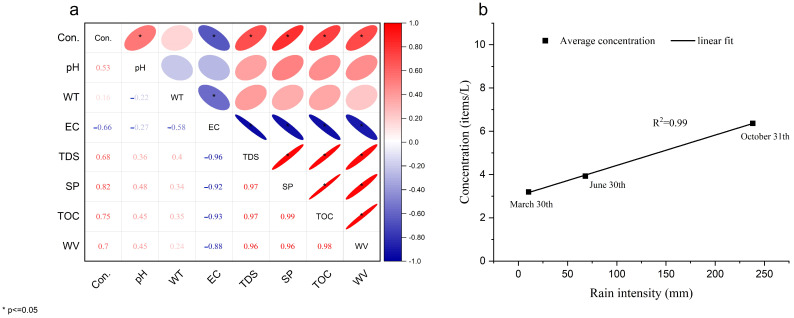
Relationship of microplastic abundance with water physicochemical properties and rain intensity. (**a**) water properties and (**b**) rain intensity.

Environmental MPs are divided into native MPs and secondary MPs; secondary MPs are the main source of MPs environment, so environmental conditions are an important factor in MP distribution and migration. The distribution and migration of water environments are affected by a variety of factors, mainly including pollution sources, terrain, hydrodynamic conditions [58], and atmospheric settlement [20]. The results of this study show that, except for the S5 sampling site, the abundance of MPs in the underground karst river gradually decreased with water flow. In contrast, the S5 sampling site was a lake formed at the underground river outlet, and large amounts of microplastics had settled there from the atmosphere. Previous studies have shown that the physicochemical properties of water may be a factor affecting the migration of MPs. Previous studies indicated that the longitudinal migration of MPs is accelerated by water temperature, salinity or biological forces, and other external forces [59]. However, some studies have also shown that the distribution and migration of MPs are not significantly correlated with water indicators, such as pH, temperature, salinity, suspended matter particles, and dissolved oxygen, which may be due to source and topography differences in water samples [60]. The correlation analysis of the karst groundwater MPs and main environmental factors by Pearson analysis showed that the karst water MPs were closely related to the water SP, TOC, and WV, with a significant correlation higher than 0.70 at *p* ≤ 0.05, as shown in Figure 8a. The suspended particles in karst groundwater are related to rainfall. Meanwhile, the concentration of suspended particles in an underground karst river rapidly increases under rainfall conditions, which is an important factor affecting the movement of substances in groundwater [61]. Microplastic properties are also important factors affecting the migration of MPs.

A previous study found that the size of MPs affects their migration in the soil and groundwater [24]. Large-sized MPs in the soil may be trapped through soil filtration, resulting in smaller sizes in the groundwater environment [62]. The particle size of MPs negatively correlates with the diffusion distance of MPs in the direction pf flowing water, whereas MPs with neutral particle size will selectively move to the shore [63]. Meanwhile, small-sized MPs are more effectively retained in rivers, and their migration is mainly affected by hydrodynamic forces, whereas MPs with a large particle size are easier to retain in water sediments [64]. Environmental conditions are also the main causes of microplastic distribution and migration. The wind has been found to play a significant role in the dispersal of MPs [65]. The wind will change the distribution of MPs settling in the water, thus affecting the migration of MPs in groundwater. Meanwhile, the study pointed out that wind power intensifies the fragmentation of floating MPs and promotes their migration. Environmental factors, such as precipitation and temperature, have been shown to alter the vertical distribution of MPs and, in turn, their migration via water [66]. This study also showed that rainfall time significantly changed the distribution of MPs in karst groundwater. The number of MPs in groundwater in the rainfall concentration (June) was twice as much as that in March. The abundance of MPs was significantly correlated with rainfall intensity, as shown in Figure 8b. In addition, our study showed that the concentration of MPs in karst underground rivers showed a certain correlation with human flow density, and the concentration of MPs at sampling sites with higher human density was higher than that of water samples at other sampling points, which is also consistent with [42].

The diameter of the bottom particle is inversely proportional to the rate at which MPs settle in river water. This may be because a smaller bottom particle size leads to less friction with the MPs in the water, thus depositing more MPs [67]. The results of this study showed that the increased rate of water flow increased the lateral migration of MPs in karst groundwater; the water flow has two positive and negative effects on the distribution and migration of MPs. First, an increase in water flow rate increases the migration of MPs, which may dilute the abundance of MPs in the water, which is consistent with Chen’s study [68]. On the other hand, an increase in water velocity may be accompanied by increases in water-suspended particles, thus holding more MPs. According to the author’s analysis, karst cracks play a significant role in the migration of microplastics beneath the karst region’s surface rivers. When soil erosion is minimal, the soil in the aquifer cracks will filter out exceptionally large particles of microplastic, allowing only those of smaller size to enter the aquifer.

### 4.3. Perspective of Ecological Risks of MPs in Karst Groundwater

Microplastic abundance and distribution of sampling areas can change ecological risk levels. This study’s results show some differences in the risk index *H* and *RCR* values at different sampling times, which is similar to the *RCR* results of the Huangpu River study by [69]. Groundwater in karst areas is an important source of drinking water for residents, and water quality is related to human health and aquatic ecosystem safety. Our study shows that karst groundwater is generally at low ecological risk. When environmental events such as large rainfall occur, the ecological risk of MPs in water bodies are significantly increased.

Different polymer chemical compositions are also important factors affecting the *RCR* of the ecological risk index of MPs. For example, for PVC, the PAN has a 300 times higher hazard index than the common microplastic PS [27]. It is worth noting that PAN and some rubber, such as styrene butadiene rubber production, are small but not easy to decompose, and accumulate in the water environment, which will significantly increase the ecological risk of MPs. MPs in the water can also enter the human body through the food chain. A survey of a Brazilian estuary found that *Stellifer* spp. had the highest amount of microplastic gut in the late rainy season [70]. It has been reported that fish feeding on plankton are more likely to feed on MPs, and wild fish intestines are mainly fibrous, probably due to the high deformability of fibrous microplastic that is more likely to be swallowed by fish [71]. In addition, the first occurrence of MPs in clams was in Taihu Lake, China, and the accumulation of MPs was 38 to 3810 times greater than that in sediment, indicating that benthic invertebrates may also have higher concentrations of MPs [72]. It is worth noting that there are some limitations in assessing the ecological risk of MPs by relying on the risk index *H* and *RCR*. One major reason is that MPs may effectively transport numerous toxic substances, including heavy metals, organic pollutants, and viruses, into the environment. Therefore, to study the ecological risk of MPs in water bodies, it is necessary to jointly consider the degree of risk of the exogenous pollutants they carry. The carrying capacity of karst groundwater resources is high. However, due to its special environment and poor purification capacity, the water MPs in the karst area are very important to regional ecological safety and human health. Therefore, it is necessary to systematically study the occurrence, migration, and destination of MPs in karst groundwater.

## 5. Conclusions

Karst groundwater is an important regional drinking water resource, and preventing microplastic pollution is important to human health. The occurrence and migration characteristics of MPs in karst groundwater were investigated, and the abundance of microplastic pollution in karst underground rivers ranged from 4.0 to 9.23 items·L^−1^, with an average value of 4.50 items·L^−1^. The abundance of MPs decreased with water flow direction, and concentrated precipitation significantly increased the abundance of MPs in groundwater. MPs in karst groundwater were mainly composed of PS and PE that were 1~5 mm in size, in the forms of colored and transparent film and fiber. This study assessed the degree of karst groundwater risk using the risk index *H* and *RCR* values, which showed that the overall microplastic risk of karst groundwater was low, but that rainfall events could significantly increase the risk level of MPs. The Pearson correlation analysis showed that the occurrence of MPs in karst groundwater was significantly and positively correlated with SP, TOC, and WV, which also showed that the pollution of MPs in karst groundwater is closely related to regional soil erosion. The study of MPs in karst groundwater needs to explore the relationship between environmental specificity and microplastic migration. This study provides preliminary research data and a future reference for further study of karst groundwater MPs.

## Figures and Tables

**Table 1:** The physicochemical characteristics of the sampled water.

Time	Sample	pH	WT (°C)	EC (μS/cm)	TDS (mg/L)	SP (mg/L)	TOC (mg/L)	WV (m/s)
30 March 2021	T3-S1	7.68	16.21	364.84	389.14	42.27	2.04	2.13
	T3-S2	7.64	16.34	379.46	394.38	41.27	2.31	2.43
	T3-S3	7.58	16.51	368.41	428.67	36.18	2.28	2.68
	T3-S4	7.46	16.84	366.37	407.11	38.77	2.61	2.48
	T3-S5	7.52	17.84	369.48	396.43	44.15	2.57	2.37
	Aver. *	7.58	16.75	369.71	403.15	40.53	2.36	2.42
	SD	0.08	0.59	5.13	14.04	2.78	0.21	0.18
	CV (%)	1.05%	3.50%	1.39%	3.48%	6.86%	8.84%	7.35%
30 June 2021	T6-S1	7.62	17.24	214.36	553.83	254.91	14.41	6.14
	T6-S2	7.66	17.46	206.57	564.81	224.65	12.44	6.24
	T6-S3	7.68	17.94	201.43	559.94	216.24	15.36	6.67
	T6-S4	7.56	17.12	216.75	581.06	210.66	13.58	6.35
	T6-S5	7.77	18.1	211.04	574.47	265.48	15.1	6.29
	Aver.	7.66	17.57	210.03	566.82	234.39	14.18	6.34
	SD	0.07	0.39	5.49	9.81	21.79	1.07	0.18
	CV (%)	0.91%	2.19%	2.62%	1.73%	9.30%	7.52%	2.84%
31 October 2021	T10-S1	7.54	18.01	268.93	481.55	116.94	6.34	3.15
	T10-S2	7.43	18.21	263.28	483.18	110.26	6.68	3.24
	T10-S3	7.51	18.33	265.84	471.26	98.54	6.04	3.57
	T10-S4	7.46	18.54	267.18	465.39	96.38	6.47	3.34
	T10-S5	7.68	18.17	260.87	486.91	123.67	6.31	3.26
	Aver.	7.52	18.25	265.22	477.66	109.16	6.49	3.31
	SD	0.09	0.18	2.85	8.03	10.47	0.47	0.14
	CV (%)	1.15%	0.97%	1.08%	1.68%	9.59%	7.26%	4.30%

Note: WT—water temperature. EC—electrical conductivity. TDS—total dissolved. SP—suspended particles. TOC—total organic carbon. WV—water velocity. * Means the average of five sample at same time. SD—standard error. CV—coefficient variation.

**Table 2:** A risk index level of MPs.

Item	0 < *H* ≤ 1	1 < *H* ≤ 10	10 < *H* ≤ 100	100 < *H* ≤ 1000	*H* > 1000
Grade	I	II	III	IV	V

**Table 3:** Risk characterization ratio (*RCR*) ecological risk rating of MPs.

Item	0 < *RCR* ≤ 1	1 < *RCR* ≤ 10	10 < *RCR* ≤ 100	*RCR* > 100
Grade	No significant risk	Slight ecological risk	Moderate ecological risk	Serious ecological risk

**Table 4:** Distribution of the microplastic at the sampling site.

Time	Site	Volume (L)	P1 (Items)	P2 (Items)	P3 (Items)	Total (Items)	Concentration (Items·L^−1^)
30 March 2021	T3-S1	2	8	7	8	23	3.83 ± 0.94
	T3-S2	2	8	5	5	18	3.00 ± 2.83
	T3-S3	2	6	6	4	16	2.67 ± 1.89
	T3-S4	2	6	5	3	14	2.33 ± 2.49
	T3-S5	2	10	8	7	25	4.17 ± 2.49
	Total MPs and average concentration at all five sites					96	3.20 ± 2.13
30 June 2021	T6-S1	2	20	18	19	57	9.50 ± 1.63
	T6-S2	2	12	11	14	37	6.17 ± 2.49
	T6-S3	2	8	10	12	30	5.00 ± 3.27
	T6-S4	2	8	12	6	26	4.33 ± 4.99
	T6-S5	2	16	10	15	41	6.83 ± 5.25
	Total MPs and average concentration at all five sites					191	6.37 ± 3.53
31 October 2021	T10-S1	2	12	10	6	28	4.67 ± 4.99
	T10-S2	2	8	8	7	23	3.83 ± 0.94
	T10-S3	2	7	8	6	21	3.50 ± 1.63
	T10-S4	2	6	6	4	16	2.67 ± 1.89
	T10-S5	2	11	9	10	30	5.00 ± 1.63
	Total MPs and average concentration at all five sites					118	3.93 ± 2.22

## Data Availability

Not applicable.
